# Reference Values for Wristband Accelerometry Data in Children Aged 6–11 Years of Age

**DOI:** 10.3389/fped.2022.808372

**Published:** 2022-04-12

**Authors:** Astrid E. Lammers, Anna Lena Romanowski, Helmut Baumgartner, Gerhard-Paul Diller, Anselm Uebing

**Affiliations:** ^1^Department of Cardiology III - Adult Congenital and Valvular Heart Disease, University Hospital Muenster, Muenster, Germany; ^2^Department of Paediatric Cardiology, University Hospital Muenster, Muenster, Germany; ^3^Department of Paediatric Medicine, University Hospital Muenster, Muenster, Germany; ^4^Department for Paediatric Cardiology, University Hospital Kiel, Kiel, Germany

**Keywords:** exercise, accelerometry data, reference value, Fitbit, child

## Abstract

**Objectives:**

Wristband activity trackers (accelerometers) could serve as a convenient monitoring tool to continuously quantify physical activity throughout the day. We aim to provide reference values for the use of these devices in healthy children.

**Methods:**

Children were recruited at a local school and provided with activity trackers (Fitbit Charge 2). Pupils were instructed to wear devices during all normal daytime activities over a period of 11–15 days. Demographic data, total number of daily steps and heart rate were recorded. In addition, all children/parents were asked to complete a questionnaire providing information about daily physical routine (mode of transport to school, sporting activities as well as sport club memberships).

**Results:**

Three hundred two children (54.6% boys; median age 8.7 years) participated in this prospective study. Median wearing time of the device was 12.1 h/day. Overall, the median daily total step count was 12,095. Median step counts/day were significantly higher in boys compared to girls (13,015 vs. 11,305 steps/day; *p* < 0.0001). In addition, step counts were significantly higher during the week, compared to weekend days. The effect of age on daily step count was found to be non-linear: the total daily step count increased from 6 to 8.5 years of age, while older children (aged >8.5 years) had lower step counts compared to the younger children. Significant predictors of the daily step count were male gender (+1,324.9 steps, *p* = 0.0008), mode of transportation to school (walking, bicycle, scooter: +865.5 steps *p* = 0.049), active membership in a sports club (+1,324.9 steps/day, *p* = 0.0008), and number of structured units of physical exercise performed (+336.5/per 45 min, *p* < 0.0001). Severe obesity was associated with a significant reduction in total daily step count (−3037.7 steps/day, *p* = 0.015).

**Conclusion:**

Our prospective cohort study of healthy school children provides reference values for wristband accelerometers in normal individuals. In addition, it clarifies the effect of age, body weight and lifestyle on normal daily step counts in school children. This data should be helpful to judge the degree of physical limitation of patients compared to healthy peers.

## Introduction

Assessing exercise capacity and physical functioning is of major interest for clinicians, treating patients with various chronic diseases affecting the cardiopulmonary system and, thus, physical exercise capacity (1). Classifying patients into a functional class is important for counseling and often affects treatment decisions (2, 3). However, this process relies on subjective assessment and may underestimate the true degree of exercise limitation, especially in patients with chronic disease who have adapted to their limitations (4). Additionally, patients with congenital conditions affecting physical ability to exercise may have never experienced a normal exercise capacity. Therefore, objective exercise testing modalities such as cardiopulmonary exercise testing (CPET) or the 6-min walk test have been advocated and are widely employed (3–7). While CPET represents the gold standard for the objective assessment of maximal exercise capacity and allows for the comprehensive measurement of physiologic parameters, it is technically demanding, requires specific expertise and not ubiquitously available (4, 5). Moreover, it is often not feasible in children of young age, as equipment usually demands a certain body length and coordination. Similarly, the 6-min walk test, often used to assess exercise capacity at submaximal levels in patients from school age onwards (8), has inherent limitations (6, 7).

Furthermore, an isolated test in a clinical environment may not fully reflect physical capacity and overall patient functioning in the patients' own natural environment appropriately.

We hypothesized that novel, commercially available lightweight and portable activity trackers represent an optimal tool to monitor patient exercise levels continuously during daily activities in a natural environment by tracking daily steps and assessing temporal activity levels. However, reference values for these devices in children are lacking.

We, therefore, aimed to test the feasibility of such a device and to provide normal values in healthy school children and to clarify the effect of age, body weight, and lifestyle on normal daily step counts in school children.

## Patients and Methods

This was a prospective study. Children were recruited from a primary school in an urban area (population 312,000) in Western Germany. Parents provided informed consent and children agreed to participate in the study. The study was approved by the local ethics committee (approval number 2018-076-f-S) of the University Hospital Muenster, Germany.

We excluded children with pre-existing chronic medical conditions likely to affect exercise ability and those with known mobility issues. Children were equipped with a commercial wrist accelerometer (Fitbit Charge 2 HR, Fitbit, San Francisco, CA, USA) and instructed to wear the device throughout the study period of 14 days during the entire daytime. Children were not excluded from the study if they did not meet a pre-defined daily wear time. The daily wear time, in turn, was assessed by inspecting the missing registered heart rate data of the device at the time of analysis. For convenience and to improve compliance, we did not request to wear the accelerometer at night. After the study period, devices were collected and the recorded data analyzed. The device digitally records steps and heart rate information every minute, thus allowing to analyze the device wearing time as well as deriving the circadian activity profile in addition to the total daily steps. Data were extracted from the devices using a custom written script, collecting and analyzing cumulative daily steps as well as minute by minute step data. In addition, the devices provide mean heart rate on a minute-by-minute basis. Furthermore, demographic information, weight, height, gender, and ethnic background were documented.

### Questionnaire Data

Parents/guardians were asked to complete a questionnaire together with their child, collecting information on daily habits of physical routine and school hours (morning vs. morning plus afternoon school attendance). While mandatory school hours are between 8 a.m. and midday, children can also voluntarily attend an afternoon school program with or without exercise activities. Information on spare time physical activities in sports clubs was collected. Structured sport activities were reported in units of 45 min. We also aimed to collect information on the pupils' routine and “unstructured” physical behavior, e.g., the mode of transport to school (walking, scooter, bicycle, bus, car, etc.). Chronic health conditions potentially affecting children's activities were enquired as well as their physical wellbeing during the tracking period. Data of children with intercurrent illnesses were excluded from the analysis. The questionnaire was developed specifically for the current study and has not been externally validated.

### Choice of the Device

The Fitbit Charge HR was chosen, since the device itself is considerably flat and after some adjustments to the armband would fit to a small child's wrist. Since this was an observational study, we switched off all possible interrogation features in the display to minimize distraction from the device itself. The children were able to see the number of steps and heart rate but did not receive any motivational tools or messages.

### Statistical Analysis

Values are presented as mean ± standard deviation or as median, 5th, 25th, 75th, and 95th centile, respectively. Interquartile ranges represent the 25th and 75th percentile. For comparisons between groups unpaired Student's *t*-test or non-parametric tests (Mann-Whitney-*U*-test) were used depending on data distribution. To evaluate associations between continuous variables we used univariate and multivariate regression analysis. As data is naturally clustered by study subject, a linear model with cluster robust standard errors was used. To assess non-linear relationships in scatterplots, the Lowess technique (LOcally WEighted Scatterplot Smoother) was used to draw a smooth line representing the average value of distance as a function of weight and height. To produce smooth non-parametric quantile regression curves for daily step counts similar to growth charts, the R *quantregGrowth* library was employed. The package allows to estimate the smooth effect of age on step counts while imposing proper constraints to prevent quantile crossing. Relevant details of the method have been discussed in detail before (9). Statistical analyses were performed using R (version 3.5.0) statistical package.

## Results

Of the 393 pupils invited to participate, 302 (77%) volunteered to take part in the study between May and September 2018. Data from four accelerometers could not be extracted; three devices were lost and one developed a technical malfunction. Therefore, we analyzed data from 298 school children [165 (54.6%) boys; age 6.8 to 11.6 years] including 4,147 subject days of tracking. The median participant age was 8.7 years (IQR 7.8–9.7 years). At the first day of data acquisition, 12 individuals (6 girls, 6 boys) were 6 years, 85 participants (41 girls, 44 boys) were 7 years, 73 participants (29 girls, 44 boys) were 8 years, 74 participants (31 girls, 43 boys) were 9 years, 51 subjects (26 girls, 25 boys) were 10 years, and 6 subjects (3 girls, 3 boys) were 11 years old.

The majority of subjects had a normal body weight (71.4%). 13.8% of participants had a body mass index below the 10th percentile, 14.8% of subjects had a body mass index >90th percentile and 5.1% of individuals were severely obese (>97th percentile) based on German age adjusted body mass index measurements (10). The median wearing time of the device per child was 14 days (range 11–15 days). Based on minute-by-minute data recording, children wore their devices for a median of 12.1 (IQR 9.4–13.9) hours per day.

### Total Daily Steps

**Figure 1:**
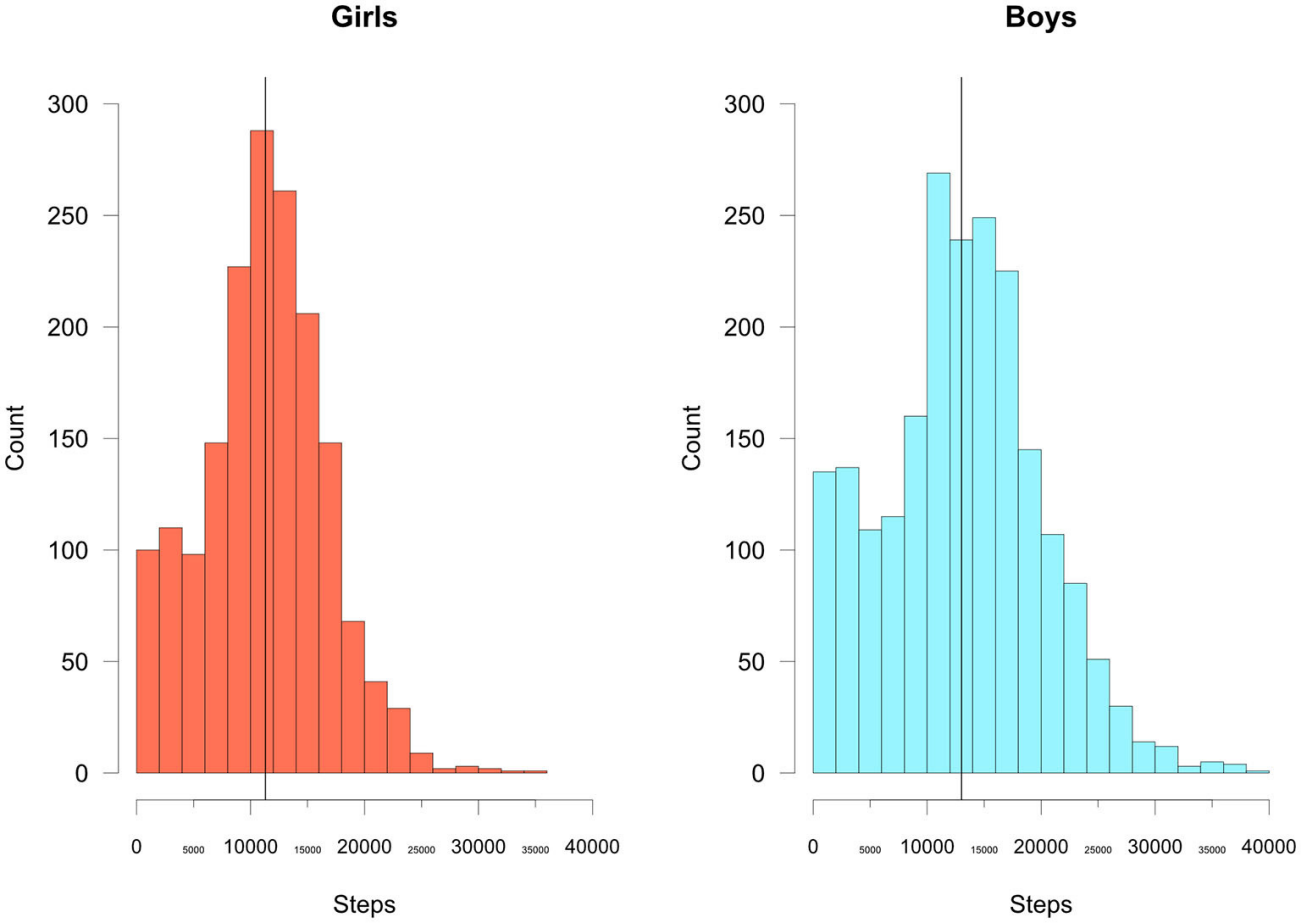
Total daily steps by gender. The vertical black line represents the median value.

**Figure 2:**
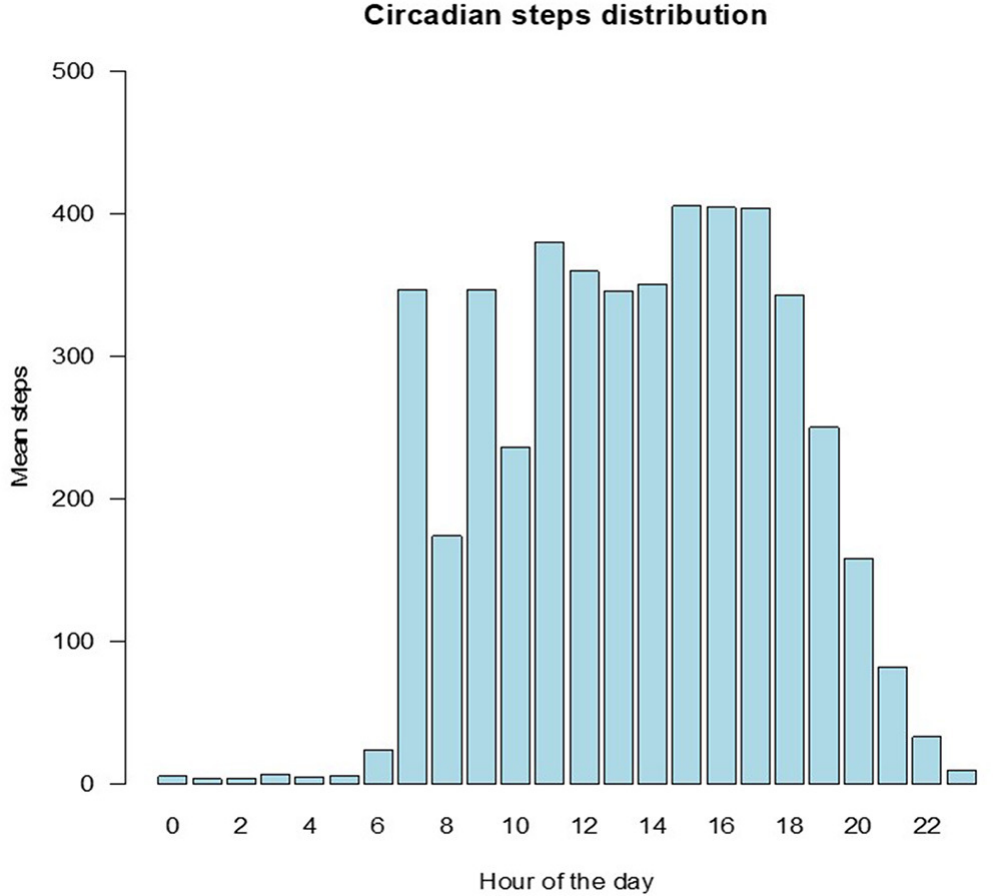
Circadian distribution of steps.

Overall, the median number of steps recorded was 12,095 (IQR 8,081–16,114). Boys were found to have a significantly higher number of steps [13,015 (IQR 8,419–17,344)] compared to girls [11305.5 (IQR 7,744–14,593), *p* < 0.0001]. Figure 1 shows the distribution of daily steps by gender. Figure 2 illustrates the distribution of steps across the day. The daily step distribution highlights peaks around the time of walking to and from school, during the typical school break times as well as during presumed playtime in the afternoon. Assessment of daily steps by day of the week showed significantly lower daily step counts on Sundays [median 8495.5 (IQR 5268.5–11283.0) steps] compared to weekdays [median 10,970 (IQR 4079.0–15129.0) steps] or Saturdays [median 10,120 (IQR 7513.5–14909.5); *p* < 0.0001 for both].

Significant predictors of total daily steps on univariate clustered linear regression analysis were male gender (effect size +1782.0 steps, *p* < 0.0001), arriving at school by foot, bicycle or scooter (effect size +865.5 steps, *p* = 0.049), active membership in a sports club (effect size +1324.9 steps, *p* = 0.0008) and number of structured sport activities (effect size +336.5/45-min unit, *p* < 0.0001). In contrast, attending school full-time (i.e., spending the afternoon supervised at school, *p* = 0.64), membership in an extracurricular school-based sports program (*p* = 0.87) and percentile of body mass index (*p* = 0.08) were not significantly related to total daily steps.

**Figure 3:**
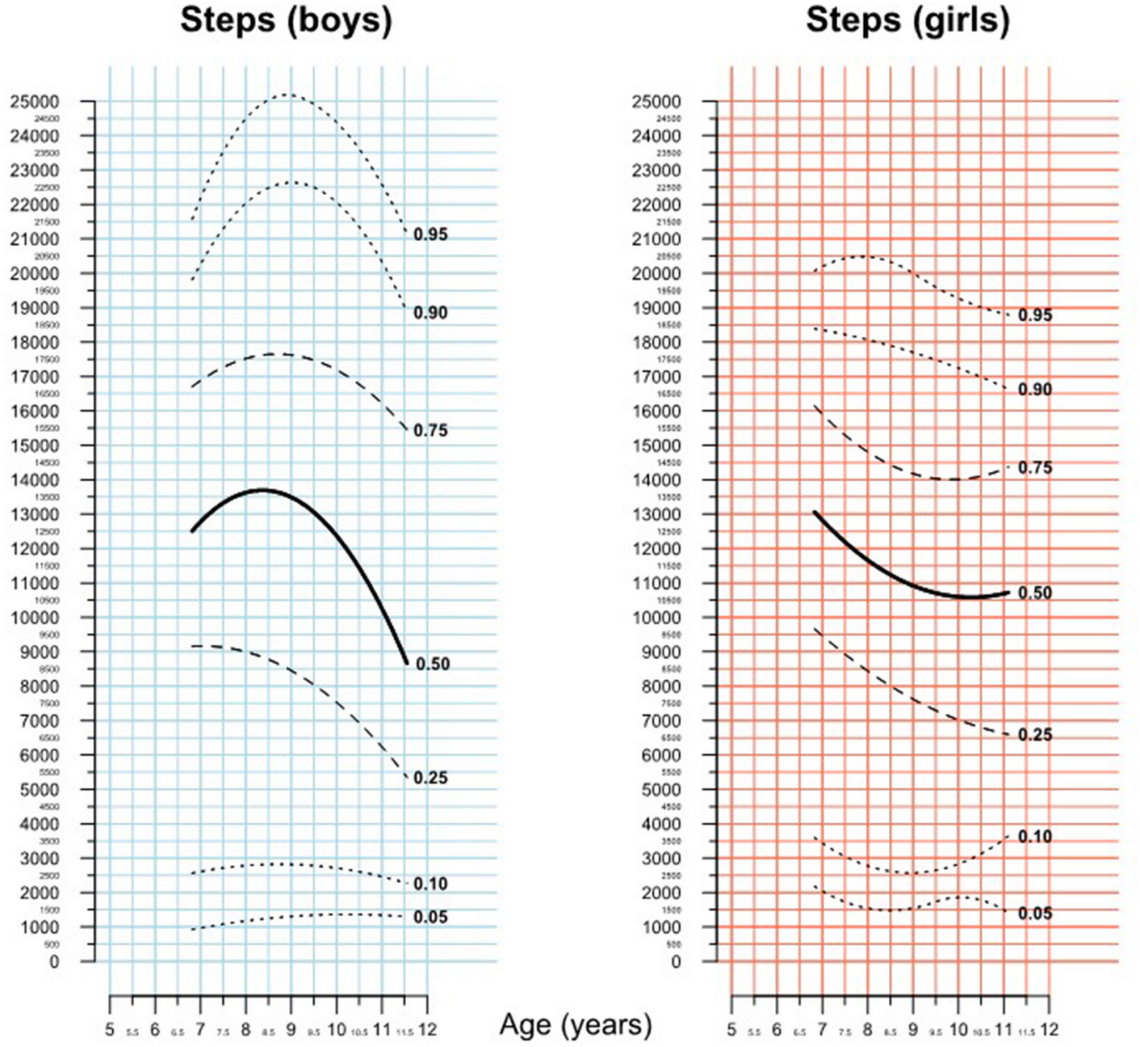
Percentiles of total daily steps.

On cluster-robust ANOVA, however, being severely obese (>97 percentile) was significantly associated with a lower total daily step count (estimate −3,037.7 steps, *p* = 0.015). As the effect of age on total daily steps was found to be non-linear on *lowess* analysis, this was not included in the above linear models. Rather the association between age and total daily steps was studied by calculating gender specific percentile curves (Figure 3). The figure illustrates a growth curve like representation with a series of percentile curves of daily steps and shows the wide variability in steps walked across all age groups. It also shows that increasing age appears to be associated with lower daily step counts, both, in boys and girls.

### Total Steps With a Daily Wearing Time of at Least 10 H

**Figure 4:**
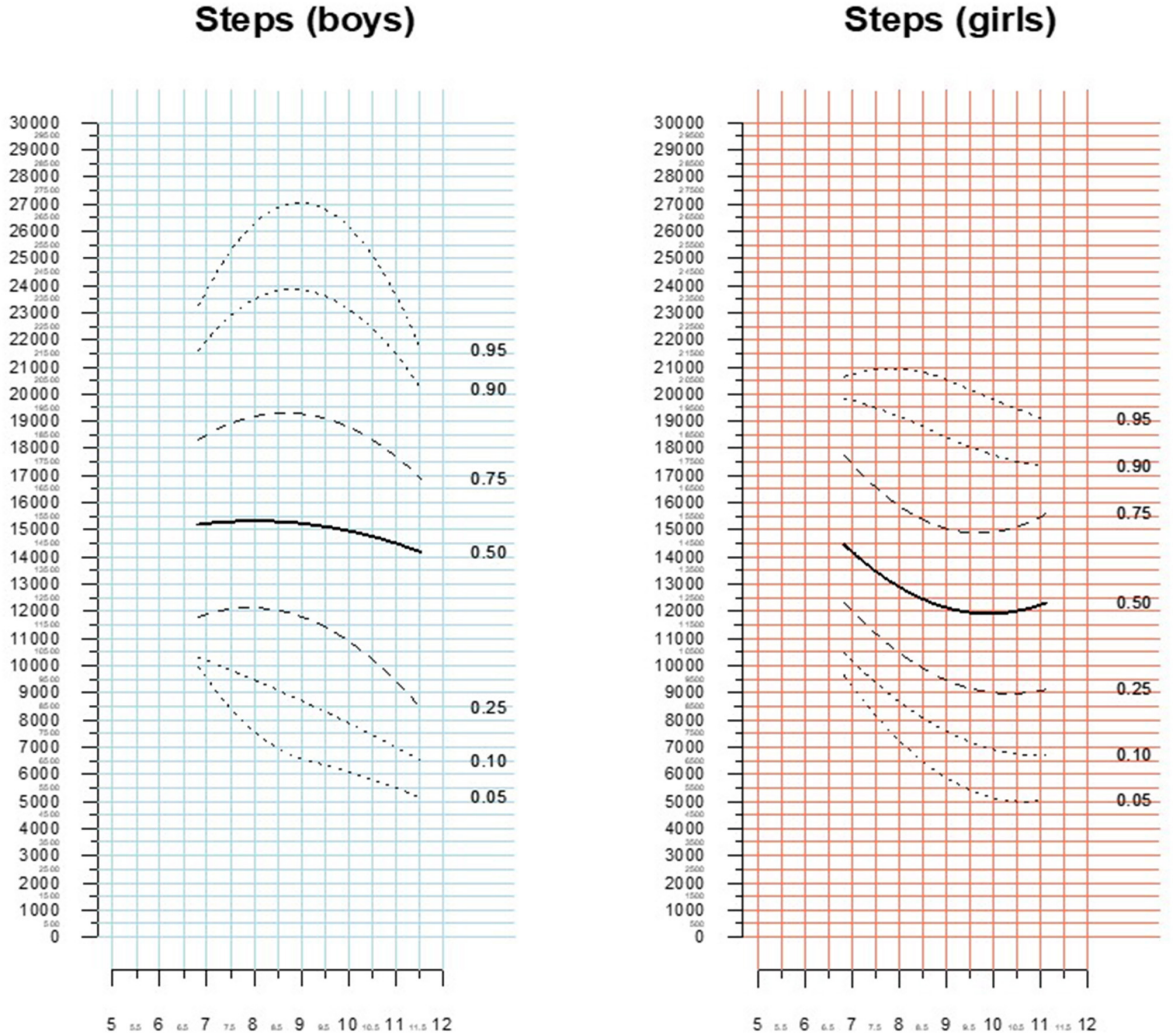
Percentiles of total daily steps (minimum wear time 10 h).

As the total daily step count is potentially affected by a limited wearing time, we also provide separate data for the patient days where the device had been actively worn for at least 10 h (*n* = 2,763). Overall, the median daily step count was 13,803 (IQR 10,700–17,345) steps in this subgroup. Again, girls had significantly lower median step counts [12597.5 (IQR 10,013–15,551) steps] compared to boys [15,220 (IQR 11,557–18,901) steps; *p* < 0.001]. Significant predictors of total daily steps on univariate clustered linear regression analysis were male gender (effect size +2592.9 steps, *p* < 0.0001), active membership in a sports club (effect size +1055.6 steps, *p* = 0.02) and number of structured sport activities (effect size +265.5/45-min unit, *p* = 0.005). In contrast, arriving at school by foot, bicycle, or scooter (*p* = 0.08) attending school full-time (i.e., spending the afternoon supervised at school, *p* = 0.39), membership in an extracurricular school-based sports program (*p* = 0.62), and percentiles of body mass index (*p* = 0.38) were not significantly related to total daily steps. Figure 4 provide percentile curves for total steps in patients days with a minimum wear time of 10 h.

### Step Counts During Leisure Time (Between 1 and 6 P.M.)

**Figure 5:**
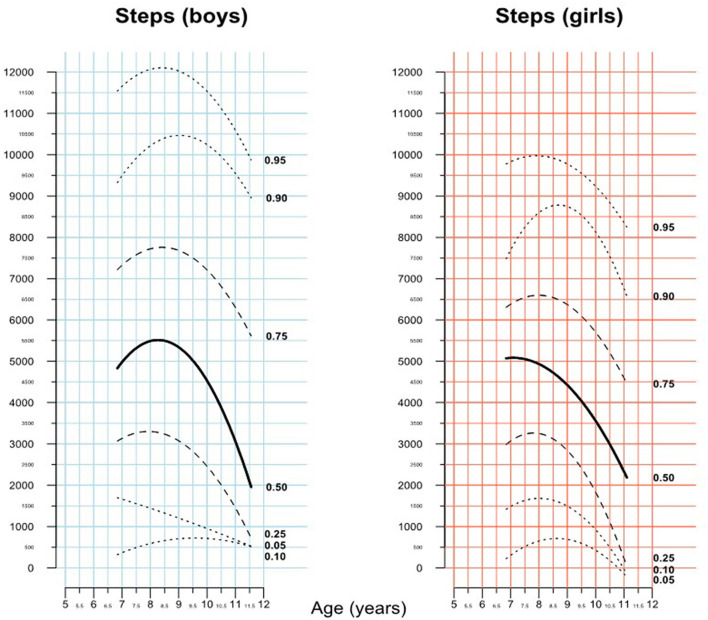
Percentiles of cumulative step counts during the time period 1–6 p.m. by gender and age group.

As children spend most of the morning time sedentary in classroom, the majority of voluntary physical activity is concentrated in the early afternoon. To capture this largely unstructured exercise we provide separate data for steps counted between 1 and 6 p.m. Overall, the median number of steps during this time period was 4682.5 (IQR 2706.5–6932.0) steps. Boys had a significantly higher step count [5055.5 (IQR 2,786–7,564) steps] compared to girls [4,244 (IQR 2,544–6,334); *p* < 0.0001]. Figure 5 shows the percentile plots for cumulative step counts between 1 and 6 p.m. vs. age stratified by gender.

### Analysis of Heart Rate Data

The median heart rate was 98.0 (IQR 90.0–105.0) beats/minute without a significant difference between boys [98.0 (IQR 91.0–105.0) beats/minute] and girls [98.0 (IQR 90.0–105.0) beats/minute; *p* = 0.99]. As expected, heart rate correlated significantly with subject age (estimate −3.57 beats per minute/year of age, *p* < 0.0001).

### Influence of Weather Conditions

The study was performed outside the school vacation period during spring and autumn (excluding the summer holidays). In Germany, there are moderate temperatures during this period. To assess the possible effect of weather conditions on physical activity levels, we performed a *post-hoc* analysis correlating daily steps with prevailing daily weather data (retrieved from an online source providing daily weather/climate data for the region of data recording - https://www.wetterkontor.de; last accessed 29/07/2021). To this end, we analyzed the effect of rainfall as categorical variable on daily step counts and found a significant reduction of daily steps by 425 steps on rainy days. Assessing days with heavy rain (defined as a rain quantity >30 L/m^2^/day), a reduction in daily steps of 5,258 steps was observed compared to the remaining days (*p* < 0.0001). Analyzing rain as a continuous variable, we found a reduction of activity of 75 steps/liter rainfall. In addition, daily step count was correlated with maximum daily temperatures with a reduction of daily steps of 130 steps/°C temperature (*p* < 0.0001).

## Discussion

To our knowledge, this is the first study to provide normal data on daily physical activity levels in primary school children and matching them with reported activities using the FitBit wristband accelerometer device. The normal data provided here should be helpful when employing wearable devices as monitoring tools in children with a chronic illness. As most schoolchildren are almost constantly in motion and physical activity primarily occurs in an unstructured way in their natural environment, we expect this type of technology-based assessment of physical capacity to be increasingly utilized in the future to supplement clinical assessment and formal exercise testing.

Evolving technology with miniaturization of recording devices will improve monitoring of physical activities during daily life and has the potential to detect subtle changes in activity patterns and activity levels. This way, the technology will help to detect any decline in physical activities potentially indicating deterioration of a medical condition early (1, 11, 12).

While temporal changes in activity patterns are patient specific, wide scale application of wearable devices in children requires information on what should be considered a normal activity pattern and thus requires reference values of daily steps in healthy age and gender matched peers. To provide this data as percentile curves was the rationale of the current study.

Reservations about the use of technology to monitor children's activity levels exist. Some parents expressed the concern that wearing accelerometers during school hours might distract their child. However, participation rate in our study was high and the feedback received from pupils, parents, and teachers after the study was excellent. After a brief period of initial excitement, children quickly lost interest in their devices, particularly since most of the active interrogation tools were switched off. Therefore, in our experience, children acted normally and where not distracted by the device. This illustrates the potential to apply this technology without significant alteration of children's behavior.

Collecting information on physical activity levels and patterns is key for the evaluation of functional ability and physical wellbeing and an essential addition to the usual clinical assessment of patients with chronic illnesses. Often, a patient's perception of its own fitness differs substantially from objective measures of exercise capacity. Therefore, most providers would supplement patient history and clinical assessment with standardized objective assessment of physical capacity (2, 4, 6, 7, 13, 14). Available tools to quantify physical capacity include 6-min walk testing, i.e., measuring the distance walked on a flat surface within 6 min (13, 14). This test also allows for the collection of physiological exercise parameters such as heart rate and oxygen saturation, before, during or after the test. The 6-min walk test is often considered to be a test assessing exercise capacity at submaximal level. It has been shown to be useful for patients, whose exercise capacity is already severely compromised. In children, the 6-min walk distance correlates well with maximal oxygen uptake from a cardio-pulmonary exercise testing and thus may reflect exercise capacity beyond submaximal levels (6, 15).

Cardiopulmonary exercise testing (CPET) is considered the gold-standard for quantification of physical capacity. In addition to maximal (or peak) oxygen uptake, it provides other valuable physiological parameters, including lung function parameters and gas exchange dynamics and provides information on the potential cause of physical compromise (15). However, CPET testing usually requires a certain body length and cooperation with the equipment used, whereas the 6-min walk test is feasible (and reproducible) from a young age (approximately 4–6 year onwards) (16). In addition, CPET requires adequate equipment and training. Both modalities absorb healthcare resources (personnel and/or equipment). Moreover, test results are influenced by multiple variables such as for instance time of the day, temperature, circumstances of testing, previous physical activities, motivation, willingness to cooperate. Therefore, test results must be interpreted with caution especially in children.

In contrast, wearable devices can provide activity data with little resource utilization, that is less influenced by motivation at a certain time point and less depend on the patient's willingness and ability to cooperate. Therefore, this method to obtain objective information on activity levels and changes over time seems particularly attractive for the monitoring and assessment of children with chronic health conditions potentially indicating disease progression. As the technology does not depend on patient cooperation it can be used in children with learning difficulties as well.

The current study is based on data acquired with the Fitbit Charge 2 device. The choice of this specific device was motivated by the relatively small size of the device making it particularly suitable for pediatric use, coupled with the ability to record data over prolonged periods of time. The device was appealing to us as it lacked GPS tracking which would contribute to faster battery depletion and could be regarded as a confidentiality concern. The device was marketed for the consumer sector at the time of study planning and has now been replaced by the Fitbit Charge 4 from the same company. Meanwhile, devices destined for children have become available.

Over time, over 750 research studies have utilized the Fitbit ecosystem with approximately only a dozen publications focusing on pediatric use of this wearable device^1^. While a modest underestimation of step counts (~160 steps/day) was reported when comparing a commercial waist worn Fitbit product against a more expensive research product in 59 healthy children, the results of the two products were found to be comparable (17). This finding is consistent with other studies in non-pediatric cohorts supporting the notion that the results obtained with various commercial products (such as FitBit or Apple Watch) and research-grade devices are comparable (18, 19).

As the aim of the current study was to assess normal values for daily steps, we deliberately deactivated interrogation of data during the data collection period. Beyond the use of wearable devices to monitor exercise patterns, these tools can be used to motivate individuals to stay physically active by presenting current exercise levels in comparison to what is considered normal or desirable and by addressing their competitive instinct. Such functions would be of interest across the spectrum of chronic pediatric diseases as well as the growing number of those afflicted by childhood obesity in developed countries.

Beyond providing normal values of step counts for wrist accelerometers, we also illustrate the circadian alterations in these measurements and correlate children's participation in extracurricular activities or sports with daily step counts. Furthermore, we show how weather conditions might affect daily step counts. These factors might explain the wide variability of normal values found in the current study. While these factors are outside the prescribing physicians' control if he or she chooses to use this type of data to monitor patients, awareness of these factors is relevant for appropriate patient counseling. Therefore, we contend that devices should be worn for prolonged periods to average out the effect of weekends or extreme weather conditions. The association between daily steps, mode of transportation to school, outside sports, and weather conditions described herewith are consistent with the results of previous studies (20–22). Moreover, healthcare professions should enquire about participation in sports activities and relevant life circumstances of patients (such as mode of transportation to school or membership in sports clubs).

The data presented here are of particular importance, when applying these devices to compromised children with a chronical illness, showing that after an initial increase of steps from 6 to 9 years, that there appears to be a decrease in number of daily steps, even in healthy children. This is important for the longitudinal follow-up of patients, implying that a potential decrease of an individual patients' step count may not be necessarily misinterpreted as consequence of disease progression.

### Limitations

The current study is based on school children recruited in a German metropolitan area. Therefore, the results are almost entirely from Caucasian children attending a state school in a high-income country. This should be considered when extrapolating the results to other geographic or economic regions. In addition, data was acquired in the spring and autumn of a region with moderate climate. As we could clearly demonstrate an impact of rain and hot weather on daily step counts and the seasonal impact on exercise capacity has been documented (22, 23) this requires consideration when applying our data to other climatic regions. Accelerometers in general may underestimate exercise levels for activities such as swimming, horse riding or especially bicycle exercise, highlighting the need to specifically enquire about this type of exercise when using these devices in clinical or research practice. The validity of the Fitbit Charge 2 fitness tracker was tested in various studies with good results, particularly for the recording of step counts (24). Slight limitations in terms of validity were found regarding the recording of heart rate during very light or very vigorous exercise (25, 26), which must also be considered when interpreting the results obtained. The current study was performed before the onset of the COVID-19 pandemic. The sub analysis of total steps with a daily wearing time of at least 10 h was based on the authors' interpretation that this might be a reasonable cut-off value to use and has no particular physiologic background. It remains unclear if and how the restrictions during the pandemic might affect normal values in healthy children. We contend, however, that with easing restrictions and return of normality in future the results presented here will continue to be of ongoing clinical relevance.

## Conclusion

Our prospective cohort study of healthy schoolchildren provides reference values to assess physical activity in children with chronic disease. This data should be helpful to judge the degree of physical limitation of patients compared to healthy peers. In addition, we clarify the effect of age, body weight and lifestyle on normal daily step counts in schoolchildren.

## Data Availability Statement

The raw data supporting the conclusions of this article will be made available by the authors, without undue reservation.

## Ethics Statement

The studies involving human participants were reviewed and approved by Ethics Committee University Hospital Muenster, Germany. Written informed consent to participate in this study was provided by the participants' legal guardian/next of kin.

## Author Contributions

AL, G-PD, and AR wrote the manuscript and analyzed the data. AL and G-PD performed statistical analysis. AL, AU, and HB supervised the process and obtained funding. All authors were involved in the critical review of the manuscript and provided intellectual input. All authors contributed to the article and approved the submitted version.

## Funding

This research was supported by the Stiftung Kinderherz Germany.

## Conflict of Interest

The authors declare that the research was conducted in the absence of any commercial or financial relationships that could be construed as a potential conflict of interest.

## Publisher's Note

All claims expressed in this article are solely those of the authors and do not necessarily represent those of their affiliated organizations, or those of the publisher, the editors and the reviewers. Any product that may be evaluated in this article, or claim that may be made by its manufacturer, is not guaranteed or endorsed by the publisher.

## Footnotes

^1^ https://www.fitabase.com/research-library/
