# Supplementary figures and images for: Reference Values for Wristband Accelerometry Data in Children Aged 6–11 Years of Age
Source: Front Pediatr. 2022 Apr 12;10:808372. doi: 10.3389/fped.2022.808372 (PMC9039362; doi:10.3389/fped.2022.808372)

**Heart rate data (Appendix A):**

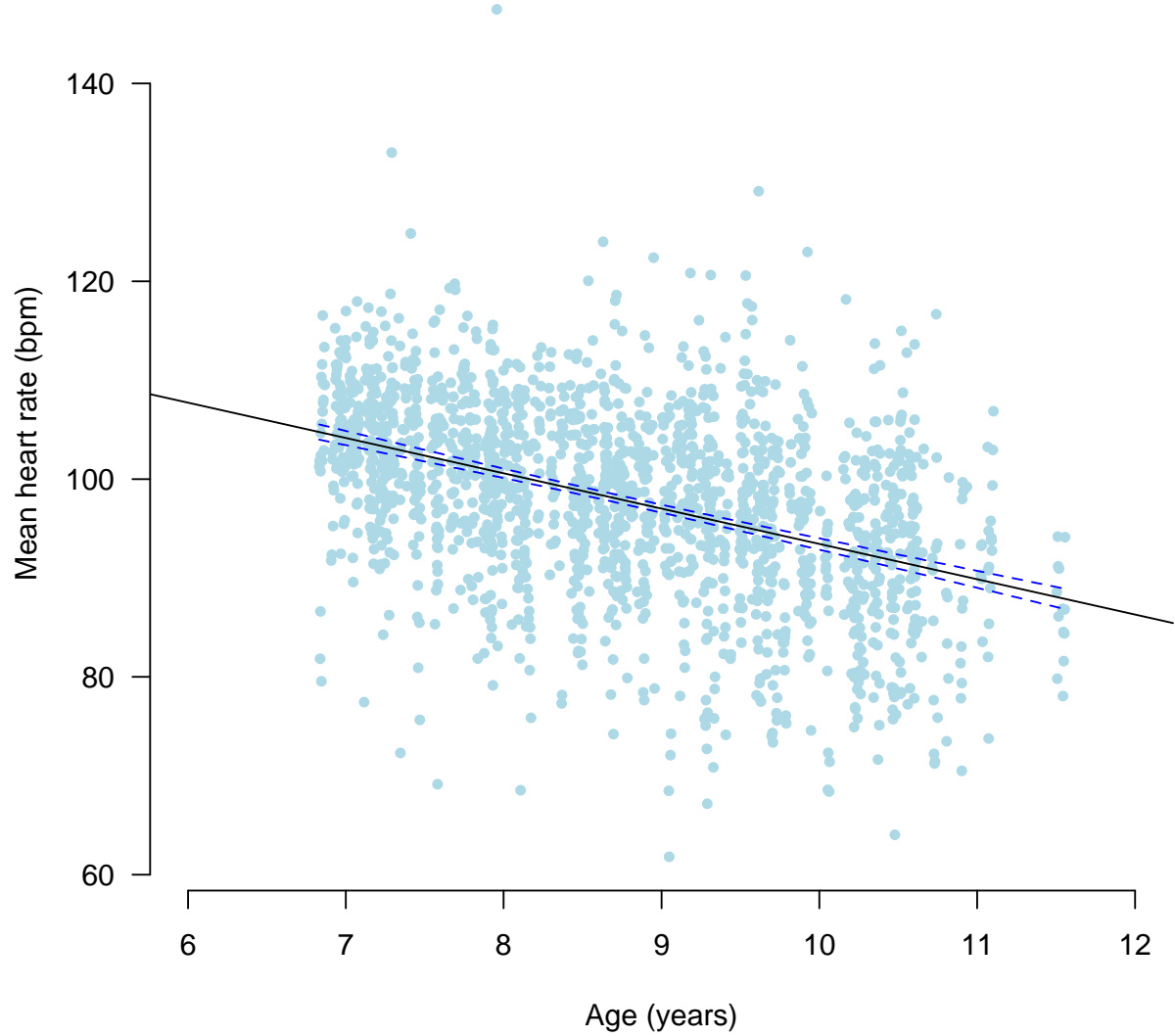

Supplement: Supplementary file 1 [file Image_1.pdf]
